# Long sedentary time is associated with worsened cardiometabolic risk factors among university employees in Eastern Ethiopia

**DOI:** 10.1038/s41598-022-26762-2

**Published:** 2022-12-23

**Authors:** Aboma Motuma, Tesfaye Gobena, Kedir Teji Roba, Yemane Berhane, Alemayehu Worku

**Affiliations:** 1 School of Nursing and Midwifery, College of Health and Medical Sciences, Haramaya University, P.O. Box: 235, Harar, Ethiopia; 2 Department of Environmental Health Science, College of Health and Medical Sciences, Haramaya University, Harar, Ethiopia; 3 Department of Epidemiology and Biostatistics, Addis Continental Institute of Public Health, Addis Ababa, Ethiopia; 4 Department of Epidemiology and Biostatistics, School of Public Health, Addis Ababa University, Addis Ababa, Ethiopia

**Keywords:** Biomarkers, Cardiology, Diseases, Health occupations, Medical research

## Abstract

Sedentary time is associated with increased risks of detrimental health outcomes. Prolonged sedentary time associates with cardiometabolic risk factors and increased mortality regardless of physical activity. Therefore, the purpose of this study was to examine the associations of sedentary time and cardiometabolic risk factors among university employees in Eastern Ethiopia. A cross-sectional study was conducted among 1200 participants. Data were collected using the World Health Organization STEPS survey instrument, and sedentary behavior questionnaire in hour per day. Sedentary time is the time spent for any duration (minutes per day or hours per day) by considering a local context. Study participants were asked how many minutes or hours they spent in sedentary time at work, their leisure time and in transportation. Finally, the total sedentary time was calculated by the sum of the individual spent in sedentary time at work, leisure, and transportation. Cardiometabolic risk factors were assessed with blood samples analysis and anthropometric measurements. The associations between sedentary time and cardiometabolic risk factors were examined using linear regression models. An adjusted coefficient (β) with the 95% confidence interval (CI) was used to report the results. *p* value < 0.05 was considered for statistical significance. The mean age of the study participants were (35 ± 9.4 years). Almost half of the study participants, 566 (48.6) were women and 598 (51.4%) were men. As the total sedentary time was increased by one unit, the body mass index increased by β = 0.61; (95% CI 0.49–0.71),waist circumference increased by β = 1.48; (95% CI 1.14–1.82), diastolic blood pressure increased by β = 0.87; (95% CI 0.56–1.18), systolic blood pressure increased by β = 0.95; (95% CI 0.45–1.48), triglycerides increased by β = 7.07; (95% CI 4.01–10.14), total cholesterol increased by β = 3.52; (95% CI 2.02–5.02), fasting plasma glucose increased by β = 4.15; (95% CI 5.31–4.98) and low-density lipoprotein cholesterol increased by β = 2.14; (95% CI 0.96–3.33) with the effects of other variables maintain constant. These findings depict the need for strategies that policymakers should promote physical activity and encouraging the breaking up of prolonged sedentary time to reduce cardiometabolic risk factors among university employees in Ethiopia.

## Introduction

Sedentary behavior refers to an energy expenditure ≤ 1.5 metabolic equivalents while in a sitting or reclining posture during waking hours and not simply the absence of physical activity^1^.Various studies have shown that, majority of university employees are spent their waking time in sedentary time^2^. For example, in high-income countries, recent evidence show that sedentary time become a public health concern with a significant risk factors for non-communicable diseases (NCDs) in employees^1,3,4^. For instance, employees in many high-income countries spent about 50% to 66% of their work time in sitting, leisure sedentary activities^5^, which become the major cause of mortality irrespective of the regular practice of physical activity^6^. In addition, evidence showed that the mean of sedentary time was about 13.4 h per day in sub-Saharan African countries in office workers in northern Ethiopia^7^.

Today cardiometabolic risk factors is one of the public health problem and the incidence of cardiovascular diseases (CVD), and type 2 diabetes^8^. Cardiometabolic risk factors, such as obesity, hyperglycemia, hypertension, and dyslipidemia become the leading causes of premature mortality^9^. In addition, evidence has shown that cardiometabolic risk factors and behavioral risk factors contribute for progression of NCDs^10^. For instance, among modifiable risk factors such as sedentary behavior importantly contributes to the development of cardiometabolic disease^11^. A recent research evidence suggested that sedentariness is an independent associated with cardiometabolic diseases^12^ like obesity^3^, raised waist circumferance^13^, and risk for type 2 diabetes and cardiovascular diseases^8^, and dyslipidemia^14^, as well as increased risks for all cause of premature mortality^3^ in employees.

Today in low-income countries, like African employees are at risk for sedentary time within change of working environments^7^. Increased technology and labor-saving devices have led to changes in employees lifestyle with prolonged desk-based and reduced activity^7,15^. Therefore, university employees, especially office workers, are become the risk population for sedentary time^7,16,17^. However, despite high sedentary time, there is a paucity of research that examines the association between sedentary time and cardiometabolic risk factors among employees in sub-Saharan Africa.We explored the associations of sedentary time in relation with different context across sedentary time domains (occupation, leisure time) with cardiometabolic risk factors in a population with a wide range of physical activity levels. Hence, identifying the association between sedentary time and cardiometabolic risk factors has great implications for evidence-based health policy and helps to design an effective intervention. Therefore, in this study, we determine the association between sedentary time and cardiometabolic risk factors among university employees in eastern Ethiopia.

## Methods

### Study settings

This study is part of a larger study on metabolic syndrome and NCD risk factors among university employees in Haramaya University, Eastern Ethiopia. Haramaya University is the second oldest university situated in East Hararghe Zone, Eastern Ethiopia. During the study period, the university has nine colleges, one academy, and one institute engaged in teaching, research, and community services with an overall of 7176 employees. The majority of the employees were males (71.9%) and administrative staff (77.9%). The study was conducted among employees randomly selected from nine colleges, one institute, and one academy from December 2018 to February 2019.

### Study design, population and sampling

The study was conducted cross-sectional study design using STROBE checklist to improve the quality of the study. The source of population was between 18 and 60 years of age in the university employees while employees who stayed in the university for at least six months during the study period was the study population. Pregnant women, critically ill, and those who self-report with some type of physical disability were excluded because unsuitability for anthropometric measurement, affect body mass index due to the increment of weight with pregnancy. We also excluded these who are on the study leave and contract staffs in the university. The sample size was calculated using the following assumptions, the standard deviation of clustered cardiometabolic risk factors score was 0.8, with the 95% confidence interval with 5% error margin, and 10% of the nonresponse rate using single population proportions^18^. For the second objective (associated factors) we used a double proportion formula to determine the sample size for significant factors reported in the previous study by considering the mean difference of body mass index was 1.3 kg/m^2^ with the power of 80%^19^. Finally, the sample size was calculated using Open Epi 3.1, having 894, but we recruited 1200 study participants. Thus, all the eligible university employees identified from the human resource database were included in the study to get a possible maximum sample size since the population was well defined (a complete sampling frame is available). Finally, sample frame was developed from human resources payroll, and a simple random sampling method was used to select eligible study participants.

### Data collection

Data were collected by using a structured questionnaire adapted from WHO STEPwise to NCD risk factor surveillance through face-to-face interviews complemented with physical measurements and biochemical tests. A locally validated WHO STEPSwise questionnaire^20^, and self-report transcultural adapted Sedentary Behavior Questionnaire was used^1,21^. The English version of the questionnaire was translated into the local language (Afan Oromo and Amharic) which was adapted to the local culture^7^. The questionnaire was pretest at Dire Dawa university employees outside of the study area. Trained experience data collectors in the area was recruited to conduct face-to-face interview, anthropometric measurements. Study participants were appointed on the next day morning in fasting states to draw venous blood. Blood sample was obtained as per the standard operating procedures (SOP) by trained medical laboratory technicians. The overall data collection process was closely supervised by the first author and master public health holder.

### Variables and measurements

The main outcome was cardiometabolic risk factors such as BMI, waist circumference, average of systolic and diastolic blood pressures, fasting blood glucose, total cholesterol, triglycerides, HDL-c, and LDL-c. A six milliliters of venous blood sample were taken from study participants’ antecubital arm in a sitting position after eight hours overnight fasting following infection prevention procedures. The sample was directed into the sterile vacuum tube (Gel Clot Activator) and placed on the rack for 10–20 min to clot. Then it was centrifuged at 3000 revolutions per minute to extract the serum and stored at − 20° for analysis. A serum sample was used to analyze lipid profile and blood glucose at Hiwot Fana Specialized University Hospital in clinical chemistry laboratory using the Mindray BS-200 chemistry analyzer (Shenzhen Mindray Bio-Medical Electronics Co. Ltd, China)^22^.

Anthropometric measurements were carried out using standard procedures and calibrated instruments. Weight was measured with the participants bare footed and wearing light clothes using a digital weight scale and measuring to the nearest 0.1 kg. Height was measured using a stadiometer with the participant’s shoes and any hats or hair ornaments removed, and participants face away from the wall with their heels together and the back as straight as possible. The head, shoulders, buttocks, and heels should be in contact with the vertical surface with the participants looking straight ahead. Then body mass index was calculated as weight in kilogram per height in meter squared as underweight (< 18 kg/m^2^), normal (18.5–24.9 kg/m^2^), overweight (25.0 to 29.9 kg/m^2^), and obese (≥ 30 kg/m^2^) according to WHO criteria^22^. The participant’s waist circumference was measured in centimeters at the midpoint of the line between the lower margin of the last palpable rib and the top of the hip bone using an inelastic measuring tape. Blood pressure was measured after resting for at least five minutes using a validated digital measuring device (Microlife BP A50, Microlife AG, Switzerland). The measurement was carefully performed on a non-dominant hand while relaxing on a flat surface in a sitting position with the back supported. Then, three consecutive blood pressure measurements were made within five minutes interval and the last two average measurements was used for the final anlayisis^22^.

Our main exposure was sedentary time, which was assessed using the self-reporting sedentary behavior questionnaire in hours per day related with any context in sedentary activities^23^. Sedentary time was estimated for ten different activities: watching television, playing computer/ video games, sitting during eating and drinking, sitting while listening to music, sitting and talking on the phone, doing paperwork or office work, sitting and reading, sitting and playing a musical instrument or doing arts and crafts, socialization with family or friends or relatives, sitting and driving/riding in a car, bus, or train. The ten items were completed for weekdays and weekend days separately, and stratified into three domains (occupation, transportation and leisure time). Sitting during occupation consisted of doing paperwork or office work; sedentary time during transportation contained sitting and driving/riding in a car, bus, or train; and leisure time sitting consisted of watching television, playing computer/video games and sitting during eating and drinking, sitting while listening to music, sitting and talking on the phone, sitting during reading, socialization, and sitting during playing a musical instrument or doing arts and crafts^24^. Total sedentary time was based on the sum of the ten items per weekday and weekend day. The average amount of sedentary time per day was calculated by multiplying weekday estimates by 5 and weekend day estimates by 2 and dividing this by 7. Finally, an estimated total sedentary time per day was calculated by summing up the average hours for all types of sedentary activities^1,25^. We categorized sedentary time into four quartiles as quartile one (≤ 4.33 h per day), quartile two (4.34 to 5.71 h per day), quartile three (5.72 to 7.27 h per day), and quartile four (**≥ **7.28 h per day)^16^. Furthermore, the items were grouped into two domain occupational and leisure sedentary time.

General characteristics contained sex, age, level of educational, ethnicity, religion, occupation, marital status and monthly salary, medical history were collected based on the WHO STEP wise approach for NCDs surveillance in developing countries. Similarly, lifestyle factors such as smoking, alcohol drinking habits, fruit and vegetable consumption, Khat chewing and levels of physical activity were measured according to WHO STEP wise approach^22^.

### Data management and analyses

All completed questionnaires were double entered into EpiData 3.1 and analyzed using STATA 16. Variables were described using proportion, mean, and quartiles as appropriate. One-way analyses of variance (ANOVA) test were carried out to compare the mean cardiometabolic risk markers groups across quartiles of sedentary time. After checking for multicollinearity by examining variance inflation factors, multiple linear regression analysis was used to test the associations between dependent variables (a number of cardiometabolic risk factors) and main independent variables (total, leisure, and occupational) sedentary time individually. Associations between outcomes and main independent variables were tested using linear regression as all outcomes were operationalized as continuous variables and also fulfill the assumption of a linear regression model. Visual inspection of P-P plots, histograms of standardized residuals, and scatter plots of standardized residuals against standardized predicted values indicated that assumptions of linearity and residuals were normally distributed and homoscedastic were checked. There was no multicollinearity in our data. Analysis of collinearity statistics show this assumption has been met, as Variance Inflation Factors(VIF) scores were well below 10, and tolerance scores above 0.2 (statistics = 1.42 and 0.74 respectively). Bivariate analysis was done with age, sex, educational status, occupation, monthly income, marital status, physical activity, smoking, khat chewing, alcohol consumption, fruits and vegetables consumption, depression and self-report health status to see the association of independent variable with the outcome variable. Those variables having a *p* value less than 0.25 were entered into a multiple linear regression model to identify the effect of independent variable with the dependent variables upon controlling confounding factors. We run nine different multiple linear regression models to examine the associations between total, leisure, and occupational sedentary time with the number of cardiometabolic risk factors. The regression coefficient (β) along with the 95% CI was reported after adjusting for possible confounder covariates and a significant association was declared if the *p* value was < 0.05.

### Ethical approval

The study protocol was performed in accordance with the relevant guidelines and regulations. The study was approved by the Institutional Health Research Ethics Review Committee of Haramaya University, College of Health and Medical Sciences (Ref. No. IHRERC/196/2018). All study participants provided written informed consent. The identity of participants were not revealed, and an identification number was allocated.

## Results

### The mean of cardiometabolic risk factors and sedentary time

The total mean (± SD) of sedentary time was 5.9 (± 2.1) hours per day, which is range of 1.3 to 11.1 h per day. The mean (± SD) of leisure sedentary time was 3.7 (± 1.5) hours per day, and mean of occupational sedentary time was 1.9 (± 1.8) hours per day. The mean of BMI is 23.8 ± 4.3 kg/m^2^ and mean fasting blood glucose was 87.7 ± 29.6 mg/dl among study participants (Table 1).

**Table 1:** The mean of cardiometabolic risk factors and sedentary time among university employees in Eastern Ethiopia, 2019.

Variable	Mean [SD]
**Cardiometabolic risk factors**	
Waist circumference, in centimeter (cm)	86.3 ± 12.7
Body mass index, in kilogram per meter square(kg/m^2^)	23.8 ± 4.3
Fasting blood glucose, in milligram/deciliter (mg/dl)	87.7 ± 29.6
Systolic blood pressure, in millimeters of mercury (mmHg)	124.4 ± 16.6
Diastolic blood pressure, in millimeters of mercury (mmHg)	79.5 ± 10.5
Total cholesterol, in milligram/deciliter (mg/dl)	184.2 ± 51.8
Triglycerides, in milligram/deciliter (mg/dl)	148.2 ± 105.6
HDL-c, in milligram/deciliter (mg/dl)	60.6 ± 18.3
LDL-c, in milligram/deciliter (mg/dl)	104.2 ± 40.2
**Mean of sedentary time [SD]**	
Total sedentary time, hours per day	5.9 ± 2.1
Leisure sedentary time, hours per day	3.7 ± 1.5
Occupational sedentary time, hours per day	1.9 ± 1.8

### Socio-demographic and lifestyle characteristics

A total of 1200 sampled participants, 1164 participated in the study (with a response rate 97%). The mean age of the participants was 35 (± 9.4) years and ranged from 20 to 60 years. Female participants account for 566 (48.6%), and older than 35 years account for 574 (47%). Two-third of participants were non-manual workers 755 (64.9%), and have a college diploma or above education was 734 (63.5%). More than half of the participants were married 667 (57.3%). Nearly quarter of the participants 305 (26.2%) were overweight and about 108 (9.3%) were obese. Out of the total study participants, 59 (5.1%) were current smoker, and 398 (34.0%) were frequent khat chewers (Table 2).

**Table 2:** Socio-Demographic and lifestyle characteristics among university employees in Eastern Ethiopia, 2019 (n = 1164).

Variable	Frequency	Percent
**Sex**		
Female	598	51.4
Male	566	48.6
**Age in years**		
Mean of age participant [SD]	35 ± 9.4	
18–24	80	6.9
25–34	537	46.1
35–44	324	27.8
45–54	151	13.0
55–64	72	6.2
**Occupation**		
Manual worker	409	35.1
Non-manual worker	755	64.9
**Level of education**		
Primary school (1–8)	193	16.6
Secondary school (9–12)	232	19.9
College and above (12 +)	739	63.5
**Service years in the university**		
< 5 years	492	42.3
5–10 years	394	33.8
10.1–15 years	148	12.7
> 15 years	130	11.2
**Marital status**		
Single	427	36.7
Married	667	57.3
Divorced/widowed	70	6.0
**Monthly salary**		
< 2000 ETB	367	31.5
2000–4000 ETB	328	28.2
4001–6000 ETB	168	14.4
> 6000 ETB	301	25.9
**Smoking status**		
Never smoker	1033	88.7
Former smoker	72	6.2
Current smoker	59	5.1
**Khat chewing**		
No/occasional	768	66.0
Frequent	398	34.0
**Alcohol consumption**		
Never/occasional	611	52.5
Regular	553	47.5
**Level of physical activity**		
< 600 MET	571	49.1
600–2999 MET	367	31.5
≥ 3000 MET	226	19.4
Body Mass Index in kg/m^2^		
< 18.5	113	9.7
18.5–24.9	638	54.8
25–29.9	305	26.2
≥ 30.0	108	9.3

ETB, Ethiopian Birr; kg/m^2^, kilogram per meter square; MET, metabolic equivalent minutes.

### Mean cardiometabolic risk factors across quartiles of sedentary time

A one-way ANOVA was conducted to determine difference mean cardiometabolic risk factors between the quartiles of sedentary time. For instance, based on the result, the mean difference of body mass index was statistically significant between quartiles of sedentary time in one way ANOVA test (F(3,1160) = 26.7, *P* = 0.000), and mean difference waist circumference between quartiles of sedentary time ANOVA (F (3,1160) = 24.1, *P* = 0.000) was statistically significant. Moreover, a Bonferroni post-hoc test revealed that the mean difference of body mass index was statistically significant in comparison of between quartile 1 and quartile 2 sedentary time (mean difference of body mass index = 0.99, *P* = 0.025); mean difference of body mass index was statistically significant in the comparison of quartile 1 verses quartile 3 sedentary time (mean of BMI difference = 1.15, *P* = 0.006). Similarly, a Bonferroni post-hoc test show that the mean difference of body mass index was statistically significant in compression of between quartile 1 versus quartile 4, and quartile 2 versus quartile 4 with (mean of body mass index difference = 3.05, *P* = 0.000), and (mean difference of body mass index = 2.05, *P* = 0.000), respectively. Also the study show that the mean difference of body mass index across quartile 3 versus quartile 4 (mean of body mass index difference = 1.90, *P* = 0.000) was statistically significant (Table 3).

**Table 3:** ANOVA analysis show the mean difference cardiometabolic risk markers between the quartiles of sedentary time among university employees in Eastern Ethiopia, 2019 (n = 1164).

Cardiometabolic risk markers	ANOVA Result (F)	Bonferroni post-hoc test of mean cardiometabolic risk factors between quartiles of sedentary time (Mean difference, *p* value)					
		Q1 vs Q2	Q1 vs Q3	Q1 vs Q4	Q2 vs Q3	Q2 vs Q4	Q3 vs Q4
Body Mass index	26.0***	0.99, *p* = 0.025	1.15, *p* = 0.006	3.05, *p* = 0.000	0.15, *p* = 1.00	2.05, *p* = 0.000	1.90, *p* = 0.000
Waist circumference	24.1***	2.47, *p* = 0.095	2.14, *p* = 0.227	8.313, *p* = 0.000	− 0.332, *p* = 1	5.84, *p* = 0.000	6.18, *p* = 0.000
Fasting Blood glucose	39.2***	6.0, *p* = 0.080	6.0, *p* = 0.057	24, *p* = 0.000	0.0, *p* = 1.0	18, *p* = 0.000	18, *p* = 0.000
Total Cholesterol	7.3***	3.0, *p* = 1.00	10.0, *p* = 0.121	18.0, *p* = 0.000	7.0, *p* = 0.738	15.0, *p* = 0.002	9.0, *p* = 0.276
LDL	4.4*	4.8, *p* = 0.873	7.2, *p* = 0.184	11.8, *p* = 0.002	2.4, *p* = 1.0	7, *p* = 0.211	4.6, *p* = 0.997
Triglyceride	11.0***	3, *p* = 1.00	14.5, *p* = 0.567	44.3, *p* = 0.000	11.5, *p* = 1.0	41.3, *p* = 0.000	29.8, *p* = 0.004
Diastolic Blood Pressure	15.0***	1.41, *p* = 0.592	1.82, *p* = 0.200	5.552, *p* = 0.000	0.42, *p* = 1.0	< 0.001	< 0.001
Systolic Blood Pressure	8.8***	0.35, *p* = 1.00	1.14, *p* = 1.00	6.15, *p* = 0.000	0.79, *p* = 1.00	5.80, *p* = 0.000	5.01, *p* = 0.002

*p* value: ***< 0.001; **0.001–0.01; *0.01–< 0.05. Q1, quadrant 1; Q2, quadrant 2; Q3, quadarent 3; Q4, quadrant 4.

**Figure 1:**
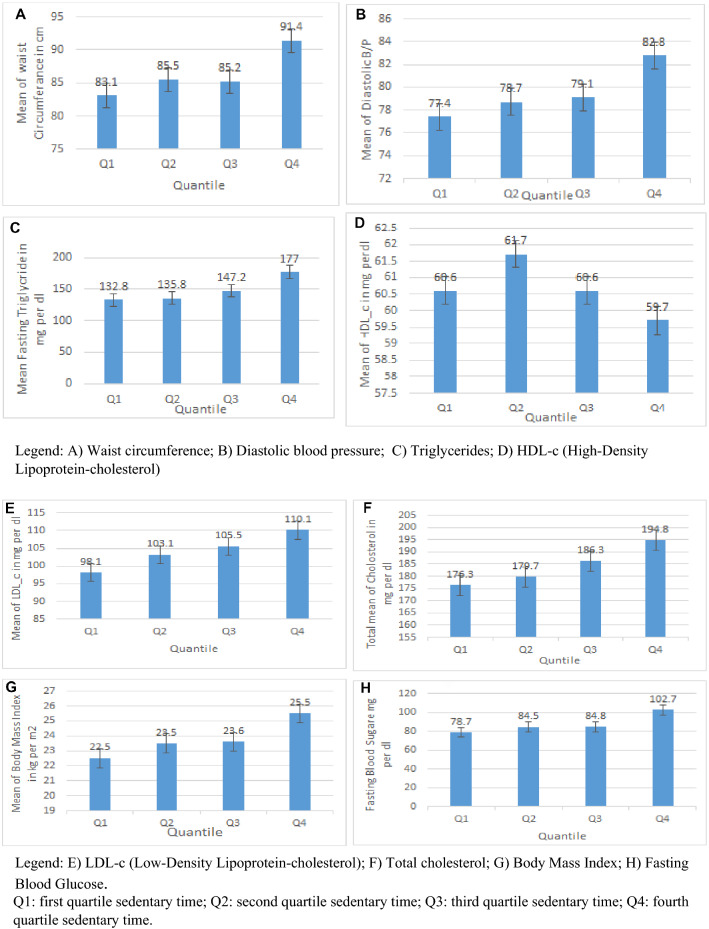
Mean cardiometabolic risk factors across the quartiles of sedentary time among university employees in Eastern Ethiopia, 2019.

The mean of waist circumference, systolic and diastolic blood pressure, body mass index, fasting blood glucose, total cholesterol, LDL-c, and triglycerides were increased within quartiles of sedentary time, however; HDL-c was decreased across the quartiles of sedentary time. For instance, as presented in Fig. 1, the mean value of triglyceride and fasting blood glucose were increased from (132.8 to 177 mg/dl), and (78.7 to 102.7 mg/dl), respectively across the quartiles of sedentary time (Fig. 1).

### Associations between sedentary time and cardiometabolic risk factors

In the final multiple linear regression model the total sedentary time and leisure sedentary time were significantly associated with cardiometabolic risk factors (*p* value < 0.05). As the total sedentary time increase by one unit, BMI will increase by 0.61 if the effects of other variables keep constant. Waist circumference score will increase by 1.48 if the effects of other variables keep constant. For a unit increase in total sedentary time, diastolic blood pressure will increase by 0.87 and systolic blood pressure will increase by 0.95 if the effects of other variables kept constant. Furthermore, one unit increase in total sedentary time, the level of triglycerides will increase by 7.07, total cholesterol will increase by 3.52, fasting blood glucose will increase by 4.15 and LDL-c will increase by 2.14 if the effects of other variables kept constant (Table 4).

**Table 4:** Associations between total, and domain-specific sedentary time with cardiometabolic risk factors among university employees in eastern Ethiopia, 2019 (n = 1164).

Sedentary time	Cardio-metabolic risk factors								
	BMI Model 1	WC Model 2	DBP Model 3	SBP Model 4	TG Model 5	TC Model 6	HDL-c Model 7	FPG Model 8	LDL-c Model 9
	β 95% CI	β 95% CI	β 95% CI	β 95% CI	β 95% CI	β 95% CI	β 95% CI	β 95% CI	β 95% CI
Total sedentary time	**0.61 (0.49, 0.72)**	**1.48 (1.14, 1.82)**	**0.87 (0.56, 1.18)**	**0.95 (0.48, 1.48)**	**7.07 (4.01, 10.14)**	**3.52 (2.02, 5.02)**	− 0.25 (− 0.82, 0.31)	**4.15 (3.31, 4.98)**	**2.14 (0.96, 3.33)**
Leisure sedentary time	**0.79 (0.63, 0.94)**	**1.59 (1.14, 2.04)**	**0.89 (0.48, 1.29)**	**0.90 (0.29, 1.52)**	**9.71 (5.68, 13.74)**	**4.01 (2.03, 5.99)**	− 0.33 (− 0.07, 0.41)	**4.75 (3.64, 5.87)**	**2.66 (1.10, 4.22)**
Occupational sedentary time	**0.28 (0.13, 0.44)**	**0.93 (0.50, 1.36)**	**0.74 (0.35, 1.12)**	**0.97 (0.39, 1.56)**	1.86 (− 1.98, 5.71)	1.59 (− 0.29, 3.47)	0.03 (− 0.67, 0.73)	**3.19 (2.12, 4.26)**	0.94 (− 0.54, 2.42)

BMI, body mass index**;** WC, waist circumferences; DBP, diastolic blood pressure; SBP, systolic blood pressure; TG, triglycerides; TC, total cholesterol; HDL-c, high-density lipoprotein-cholesterol; FPG, fasting blood glucose; LDL-c, low-density cholesterol; CI, confidence interval. Statistically significant associations (*p* < 0.05) are highlighted in bold. β- Beta coefficient. Confounders that were adjusted for the models are sex, age, service year, educational level, monthly salary, occupation, marital status, alcohol consumption, khat chewing, physical activity, smoking, self-reported health status, depression.

## Discussion

There are huge gaps of information in most African countries mainly in Ethiopia about sedentary time record or trends^7^. This is the first study attempt to estimate the associations between sedentary time and cardiometabolic risk factors among university employees in Ethiopia. The findings of this study show that sedentary time was significantly associated with cradiometabolic risk factors after adjusting for age, sex, educational status, occupation, monthly income, marital status, physical activity, smoking, khat chewing, alcohol use, fruits and vegetables consumption, depression and self-report health status. We found that the overall mean of sedentary time was 6 h per day among university employees. The result revealed that one unit increase in sedentary time and leisure sedentary time were significantly associated with BMI, fasting blood glucose, diastolic and systolic blood pressure, waist circumference, triglycerides, and LDL-c. Also one hour per day increase in occupational sedentary time was associated with BMI, fasting blood glucose, diastolic and systolic blood pressure, and waist circumference after adjustment for covariates.

In our study, we found that the mean of total sedentary time was about 6 h per day. These results highlight that sedentary time is not only present in the general population, but also highly prevalent in university employees. The finding of this study was lower than the study conducted in urban civil servants in southern nations, nationalities and peoples’ region, Ethiopia which was 13.4 h per day^7^. The discrepancy might be due to the sample size difference which was 375 in previous study, the working environment setups and types of occupation, because in previous studies participants were recruited office workers that they were more risk for sedentary time; while our study participants who work in diversity of job, ranging from high level academic work to the low level manual work. In addition, this discrepancy might be due to the study period difference, the differences in the age of study subjects, socioeconomic status, residence & lifestyle, and physical activity may contribute to the different mean of sedentary time in these different studies. Likewise, the most common and popular practice or culture in the study area are expected to participate in different social sedentary activities, which are not incorporated in this study such as groaning, social congregations, and visiting bed waiters in their spare time to khat chewing that can add to their elevated daily sitting time. Moreover, in our study it is likely to be underestimated at least to some degree due to self-reporting sedentary time. This observation suggests that public health interventions should be develop to reduce sedentary time to address university employees-wide scale.

In this study, we found that a one hour per day increase in sedentary time associated with cardiometabolic risk factors, which coincides with previous evidence^26,27^ regardless of physical activity and other potential confounders. Our findings consistent with previous cross-sectional studies^28,29^, that found a deleterious association with cardiometabolic risk factors^14,19,28–31^. Moreover, evidence showed that sedentary time associated with fasting blood glucose, triglycerides, and waist circumferences^32^. Those who sedentary for longer leisure-time like university employees whose activity is more of computer use, writing, reading usually spent much time in sedentary are at risk of cardiometabolic diseasess^23^. The typical job in the study participants often more involve non-manual labor, most individuals in our population were highly educated who typically perform desk-based office work, thus resulting in less physical activity and more sedentary time^33^. This might be contribute to abdominal obesity drives the development of cardiometabolic risks through altered secretion of adipocyte-derived active substances called adipokines, including free fatty acids, adiponectin, interleukin-6, tumour necrosis factor-alpha, and plasminogen activator inhibitor-1, and through exacerbation of insulin resistance and associated cardiometabolic risk factors^34^.This might be explain as sedentary time is associated with a reduction of lipoprotein lipase activity^35^, which reduces the absorption of plasma triglycerides and glucose uptake^36^, and increase free fatty acid in skeletal muscle and blood vessels^37,38^.

Contrary to previous studies, there was no evidence for the association between sedentary time and HDL-c^16^. This might be explained by methodological differences. For example, we assessed sedentary time of participants based on weekday and weekend by using self-reporting sedentary behavior questionnaire consisting of ten different sedentary activities on weekday and weekend. An average sedentary hours across all days were calculated using a weighted average: (weekday hours × 5) + (weekend hours × 2)/7 compared to Honda et al. which collected 28 days before data collection time^16^, and previous studies were asked questions about the number of hours they spent sitting down (cumulative sitting time)^8^. As such, we may not capture the association between sedentary time and HDL-c in our study.

Interestingly, most of sedentary time was spent during leisure time activities rather than during work. This observation in line with previous finding^1,39^. Leisure sedentary time was positively associated with BMI, diastolic and systolic blood pressure, LDL-c, triglyceride, and fasting blood glucose in line with the previous studies^10,27,28^. However, these findings are inconsistent with prior studies^40,41^.Epidemiological studies indicate mixed evidence on the association between leisure sedentary time and cardiometabolic risk factors. This might be related to the use of different measurement thresholds and diagnostic tools. Recent studies have shown that watching television and working on the computer has negative effect on cardiometabolic risk factors^18,41,42^. For example, leisure sedentary time (3 or 4 + hours) could increase the risk of cardiometabolic risks regardless of physical activity in a working adult^27^. One of the reasons for leisure sedentary time being associated with more cardiometabolic risk factors than occupational sedentary time might be concurrent health behaviors with leisure time sedentary behavior. For example, overall leisure sedentary time includes TV watching, which is often associated with snacking and as it may be associated with other unhealthy behaviors, such as greater food consumption leading to elevated risk of cardiometabolic risk factors^43^. Other unhealthy behaviors related to leisure time may explain the greater number of associations with leisure sedentary time vs. occupational sedentary time. For instance, television viewing is associated with greater risk of cardiovascular diseases and mortality compared with occupational sitting, and, therefore, reducing screen time may be the most effective target for lowering cardiometabolic risk factors^44^. Nonetheless, our observations may have important implications for leisure sedentary time interventions in university employees. Since the time spent sedentary during leisure time is significantly higher compared to occupational sitting, workplace interventions for reducing total sedentary time might have limited effects in our population. Possibly, interventions focused on reducing sedentary during leisure time (e.g. watching TV/video, socializing with friends and/ or family and computer use) may be more relevant, especially since this type of sedentary time counts for 51% of the total sedentary time in our population.

Fortunately, occupational sedentary time is not strongly associated with a set of cardiometabolic risk factors compared to leisure sedentary time, with β coefficients of (total cholesterol 1.59 vs. 4.01; triglycerides 1.86 vs. 9.71; and LDL-c 0.94 vs. 2.66) respectively. The results support the previous study findings that occupational sedentary time is less harmful to cardiometabolic risk markers than leisure sedentary time^39,42,45^. Alternatively, occupational sedentary time was derived from a single item in the questionnaire, whereas leisure time sitting was calculated from seven items. Hence, study participants may have been reluctant to score a high sedentary time on a single item. Nonetheless, our observations show that occupational sedentary time positively associated with cardiometabolic risk factors which align with previous findings waist circumference^46^, BMI^39^, fasting blood glucose, and triglycerides^47^. This inconclusive association between occupational sedentary time and cardiometabolic outcomes^48–50^. The inconsistencies may be related to different types of occupations or the amount of accumulated occupational sedentary time. These findings indicate that some, but not all, cardiometabolic risk factors correlates with occupational sedentary time, suggesting that tailored interventions may be needed to reduce sedentary time across different domains and in specific target groups.

Self-reporting leisure and occupational sedentary time appear to be associated with a number of cardiometabolic risk factors in university employees. Being an employee may be increase social connection, which can lead to long sedentary time and fewer opportunities for physical activity. Thus, our findings suggest the need for intervention that focus on reduce sedentary time to prevent cardiometabolic risk factors, early cardiovascular disease and type 2 diabetes. University employees who spent an excessive amount of time in sedentary time should be advocate the need for increasing physical activity and decreasing sedentary time through systemic intervention in and out of work programs to reduce the incidence of cardiovascular diseases.

### Strength and limitations

The strengths of this study include a large sample size and physically active individuals with a broad range of the diversity of jobs, ranging from high level academic work to the low level manual work. In addition, we used an extended questionnaire to inquire sedentary time in three domains. Data were also collected using WHO STEPS survey manual tool. It has three steps to measure socio-demographic, and behavioral related factors, anthropometric measurement and biochemical test. However, limitations of our study include self-reported data on sedentary time, physical activity and disease history, which all may cause measurement errors. This study relied on self-report data collected through interviews, which could be affected by the recall and social desirability biases. The study employed a cross-sectional study design which could not conclude causality and effects. Moreover, this finding may not be generalized to a broader Ethiopian population since our study participants were on university employee of a specific organization. Furthermore, in this study, we did not include ecological environmental constructs, organizational policy, and physical environment that could affect the sedentary time of study participants.

## Conclusions

Based on the evidence, our results show that sedentary time is highly prevalent in university employees. This study show that total and leisure sedentary time are positively associated with a number of cardiometabolic risk factors. In the study, the mean difference of cardiometabolic risk factors were statistically significant between quartiles of sedentary time. The total and leisure sedentary time are positively associated with fasting plasma glucose, BMI, systolic and diastolic blood pressure, waist circumference, triglycerides, total cholesterol, and LDL-c. Furthermore, occupational sedentary time is positively associated with fasting plasma glucose, BMI, systolic and diastolic blood pressure, and waist circumference. Therefore, these observations indicate that introducing interventions to reduce sedentary time and a regular screening for cardiometabolic risk factors is an essential to prevent the development of cardiovascular disease among university employees. These observations indicate that interventions to reduce sedentary time should incorporate domain-specific sedentary time to enhance the effect size and specifically target the most important leisure-time. We are also recommended prospective follow-up research to establish the temporal relationship.

## Data Availability

The data are available from the corresponding author upon request.
